# The association of urinary uric acid excretion with ambulatory blood pressure values in patients with chronic kidney disease

**DOI:** 10.1186/s40885-020-0136-6

**Published:** 2020-02-15

**Authors:** Ying Xu, Xun Zhou, Yuqi Zheng, Haochen Guan, Chensheng Fu, Jing Xiao, Zhibin Ye

**Affiliations:** 1 Department of Nephrology, Huadong Hospital Affiliated to Fudan University, No. 221 West Yan’an Road, Shanghai, 200040 People’s Republic of China; 2 Shanghai Key Laboratory of Clinical Geriatric Medicine, No. 221 West Yan’an Road, Shanghai, 200040 People’s Republic of China

**Keywords:** Ambulatory blood pressure, Chronic kidney disease, Hypertension, Urinary uric acid

## Abstract

**Background:**

To analyze the association between hypertension and urinary uric acid excretion in patients with chronic kidney disease (CKD).

**Methods:**

We screened 87 patients who had been admitted at the Dept of Nephrology, Huadong hospital between April 2017 to April 2019 who had completed 24-h ambulatory blood pressure monitoring and retained 24-h urine biochemical test specimens, thirty adult patients (age ≤ 65 years) with CKD 1–2 stages were recruited in the study. Pearson’s correlation analysis and multiple linear regression analysis were used to study the correlation of urinary uric acid excretion with ambulatory blood pressure values and the association of morning mean diastolic pressure (mMDP), night mean diastolic pressure (nMDP) and CV of dMSP (coefficient of variation of day mean systolic pressure) with fractional excretion of uric acid (FEua) and uric acid clearance rate (Cur). Independent T test was used to compare the differences of blood pressure values in FEua1 (FEua< 6.0%) and FEua2 (FEua≥6.0%) or Cur1 (Cur < 6.2 ml/min/1.73 m^2^) and Cur2 (Cur ≥ 6.2 ml/min/1.73m^2^) groups according to the median of FEua or Cur, respectively.

**Results:**

After adjusting for confounding factors, multiple linear regression analysis showed that FEua was positively associated with the mMDP and nMDP, Cur was positively associated with CV of dMSP. Levels of mMDP and nMDP in FEua1 group was lower than that in FEua2 group (both *P* < 0.05), level of CV of dMSP in Cur2 group were higher than that in Cur1 group (*P* < 0.01).

**Conclusions:**

We demonstrated that there is a positive correlation of FEua with morning and night mean diastolic pressure separately and Cur is positively related to CV of dMSP in CKD population. Monitoring the trend of urinary uric acid, may have a role in the early detection for hypertension or relative risks in the population of CKD.

## Introduction

Hypertension (HTN) is quite prevalent in chronic kidney disease (CKD) and is the leading cause of death. The important characteristics of hypertension in CKD are morning blood pressure surge (MBPS) and nocturnal hypertension. Previous studies have shown that MBPS and nocturnal hypertension may increase the risks of cardiovascular event, vascular disease and inflammation and silent cerebrovascular disease [1, 2]. The related mechanism underlying the phenomena covers several aspects, including the variation of vagal and sympathetic tone across day and night, the activation of renin-angiotensin system early in the morning, the peripheral vascular resistance and urinary sodium and potassium excretion. The variability of blood pressure is able to predict cardiovascular event, but is not associated with absolute blood pressure levels and was not regularly assessed in clinical practice [3]. In recent decades, it has been proved that increased blood pressure variability (BPV) directly obtained by 24-h ambulatory BP monitoring (ABPM) was associated with end-organ damage, cardiovascular events, and mortality [4].

Hyperuricemia is also common in CKD, with a prevalence as high as 8.4% in China [5]. We have found that urinary uric acid excretion is related to urinary sodium and potassium excretion in hypertension with CKD [6]. As is known to all, the secretion and excretion of Na^+^ and K^+^ are depended on Na^+^-K^+^-ATPase (NKA). In fact, the driving force of urate transporters in renal tubules comes from its basolateral Na^+^-K^+^-ATPase too. Furthermore, the NKA/Src complex has been shown to be activated in the kidney to address excess salt, and the tradeoff could be the development of salt-induced hypertension [7].

Therefore, we made a reasonable hypothesis that urinary uric acid excretion would lead to the abnormality of HTN in CKD. Hence, we study the association of urinary uric acid excretion with ambulatory blood pressure values. In this study, the urinary uric acid excretion indicators are measured in 24-h urinary uric acid (24-hUua), fractional excretion of uric acid (FEua), excretion of uric acid per volume of glomerular filtration (EurGF) and uric acid clearance rate (Cur).

## Materials and methods

### Study participants

**Fig. 1:**
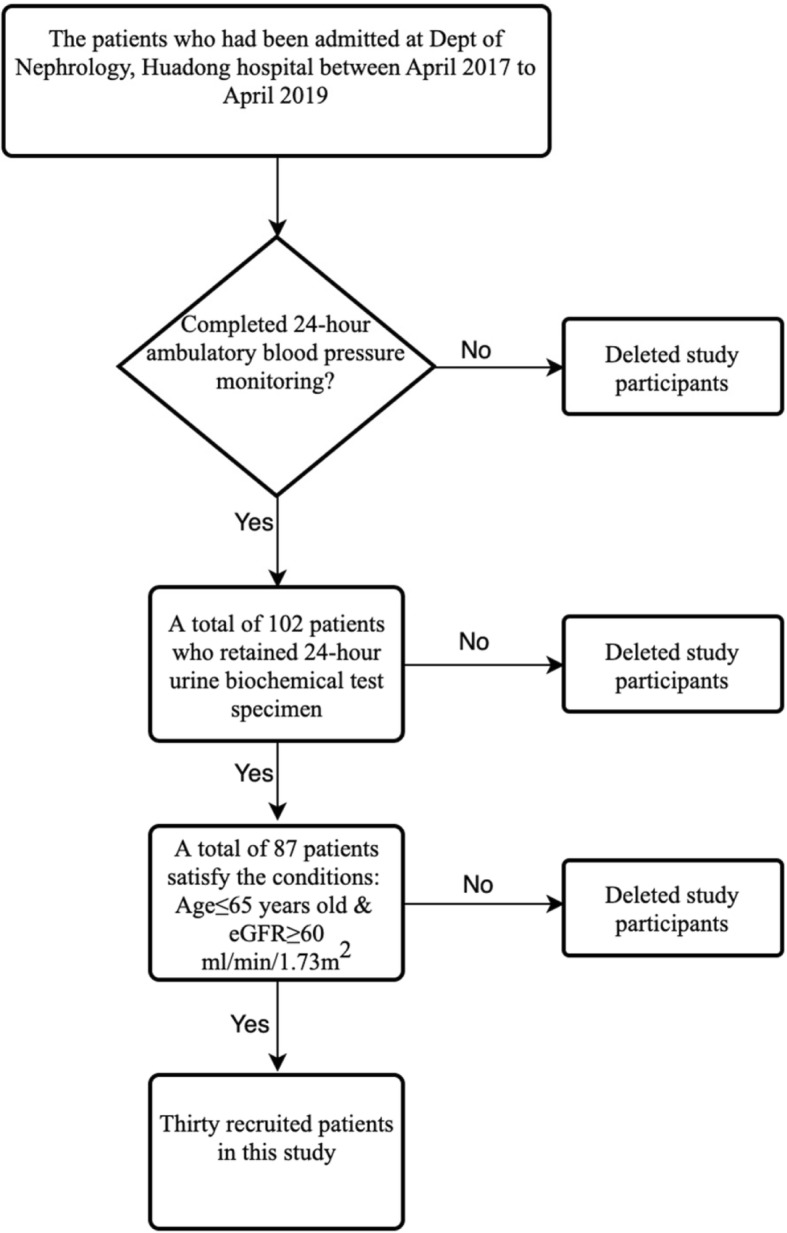
Procedures for recruiting subjects

In this cross-sectional study, a total of 87 adult patients with CKD admitted to department of nephrology in Huadong Hospital affiliated to Fudan University were collected during two-year period from April 2017 to April 2019, who had completed 24-h ambulatory blood pressure monitoring and retained 24-h urine biochemical test specimens. We eventually recruited 30 patients with two conditions: first, age should be equal or less than 65 years old; second, the damage of renal function is not serious (eGFR≥60 ml/min/1.73m^2^) (Fig. 1). The conditions for screening CKD was not limited to a single kidney damage, it covers abnormalities of kidney structure of function, present for > 3 months, with implications for health [8]. All the patient must be in a stable clinical status. In the history of medication, excluded the patients if the they take drugs that affect uric acid metabolism within 2 weeks including diuretic, losartan, aspirin, glucocorticoids, cyclosporine, immunosuppressive agents, anti-tuberculosis drugs, sodium bicarbonate, levodopa, metformin, fenofibrate and UA-lowering agents, such as febuxostat, benzbromarone or allopurinol.

### Clinical and laboratory measurements

Each participant was instructed to collect the urinary sample correctly. They were provided with a plastic bucket, following settled steps. It is noteworthy that 24-h urine collection must be done by discarding the first morning void and collecting all urine output for the next 24 h, including the first morning void the next day. Well-trained nurses are responsible for recording the start and end of each specimen collection and using a standard questionnaire for complete interviews. To determine the accuracy of a 24-h urine collection, we designed two different ways to verify. First, total amount of urinary creatinine was measured in clinical laboratory, which was used to evaluate the adequacy. Considering that all patients were in CKD 1–2 stages, level less than 14 mg/kg/day in men and 11 mg/kg/day in women indicated inadequate collection. The second method compared discrepancy between the total amount of urinary creatinine measured and calculated. The calculated value was equal to total urine volume times the concentration of urinary creatinine, the former was recorded and measured after the collection, while the latter was measured by urine left at a certain point during this admission period. Adequacy was assessed at 20% differences, such that any two values with a difference greater than the cutoffs described above would be considered inadequate. The urine collection that were judged inadequate would be traced back to the source and removed from the sample list. The two methods mentioned above were used to comprehensively evaluate the accuracy of a 24-hou urine collection.

Each sample was immediately sent to clinical laboratories for analysis of 24-h urinary levels of uric acid, creatine, sodium, potassium, glucose, albumin and urinary volume. So that we used the following indicators to estimate the excretion of uric acid and renal function. FEua was calculated as (Uur × Scr)/(SUA × Ucr) × 100, expressed as percentage. Cur was calculated as Uur × UV/SUA. EurGF was calculated as (Uur × Scr)/ Ucr. Cur and EurGF were corrected for the body surface area.

The values of blood pressure were recorded by ambulatory blood pressure monitoring. Patients are randomly assigned two kinds of models (model ABPM 6100, produced by Welch Allyn, Inc. and model ABPM-05, produced by Meditech Ltd.). The blood pressure measurement and measurement accuracy of the two machines are same, but the measurement range of them has slightly difference, with the former ranging from 25 mmHg–270 mmHg and the latter ranging from 0 to 300 mmHg, which has no effect on the results. The monitoring interval is every 20 min during the daytime and 30 min during the night time in the two machines. Ambulatory blood pressure monitoring offers a special way of automated measurements of blood pressure during a 24-h period while patients are engaged in their usual activities, including the sleep. This type of measurement is superior to standard office measurement in getting more time points’ value and more accurate data. We could obtain diverse data, such as average blood pressure, even the blood pressure coefficient of variation (CV) of each time period and all day long. As an indicator for variability in blood pressure, it is defined as CV of BP as standard deviation (s.d.) divided by corresponding average BP. Each day is divided into three periods, morning hours (6 a.m.-8 a.m.), daytime (8 a.m.-10 p.m.) and night time (10 p.m. to next 6 a.m.); average blood pressure in each period was separately abbreviated as mMSP, mMDP, dMSP, dMDP, nMSP and nMDP, for all day substituted for 24-h MSP and 24-h MDP.

After fasting for 12 h at night, each patient was taking venous blood for routine biochemical test by nurses. We could get symbols of each patient about the serum levels of uric acid (SUA), creatinine (Scr), urinary nitrogen (BUN), total cholesterol (TC), triglycerides (TG), high-density lipoprotein (HDL), low-density lipoprotein (LDL) and fasting blood glucose (FBG). The estimated glomerular filtration rate (eGFR) (milliliters per minute per 1.73 m^2^) was calculated by the Chronic Kidney Disease Epidemiology Collaboration (CKD-EPI) formula [9].

Height and weight were measured by well-trained nurses in a standard process. Body mass index (BMI) was calculated as weight in kilograms divided by height in meters squared.

### Diagnosis and grouping criteria

Hypertension was defined as the average daytime blood pressure above 135/85 mmHg, nighttime blood pressure above 120/70 mmHg, and 24-h blood pressure above 130/80mmHg [10]; in clinical practice, it is generally considered that patients who are taking antihypertensive drugs have hypertension, especially BP that has been controlled after drug therapy, regardless of whether the BP is normal or not. Hyperuricemia was defined as the level of serum uric acid above 7 mg/dl in men and above 6 mg/dl in women in line with the recommendations of an epidemiology conference held in Rome in 1963 [11], which is widely accepted and applied.

Study participants were divided into two groups according to the median of FEua (FEua1: FEua< 6.0%, FEua2: FEua≥6.0%) and Cur (Cur1: Cur < 6.2 ml/min/1.73m^2^; Cur2: Cur ≥ 6.2 ml/min/1.73m^2^).

### Statistical analysis

The continuous variables are reported in mean ± SD and categorical variables are presented in percentages. Medians with inter quartile range (IQR) are presented in case of nonparametric data. Comparisons between groups were made by Student’s t-test and one-way ANOVA. Least significance difference (LSD) test was used when the variance was homogeneous, or Tamhane’s T2 test was used. Non-normally distributed data was compared using Mann-Whitney U test. Categorical data was compared using the Chi-square test. Correlations were detected by Pearson’s or Spearman’s depending on the distribution of the data. If Pearson’s correlation analysis was statistically significant, multiple linear regression analysis was performed. Multiple linear regression analyses were performed to determine the association of urinary uric acid excretion with 24 h ambulatory blood pressure values. Statistical significance for all analyses was set at *P* < 0.05. Statistical analysis was performed with software SPSS 22.0, Stata 14.0, and Prism 7.0a.

## Results

### Characteristics of the study population

**Fig. 2:**
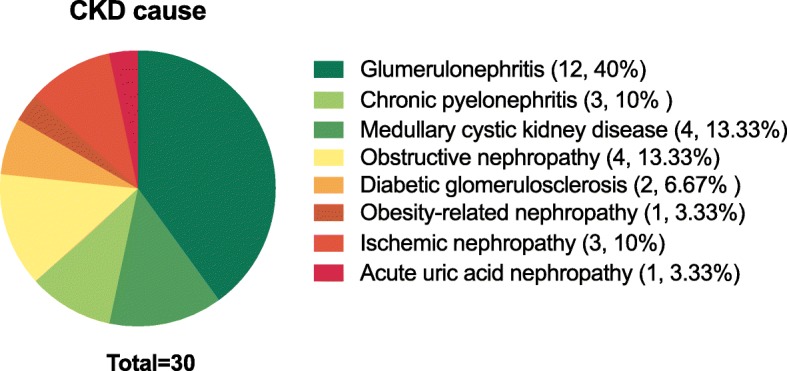
The causes of CKD. The pie chart shows the different components of renal injury in patients with CKD

**Fig. 3:**
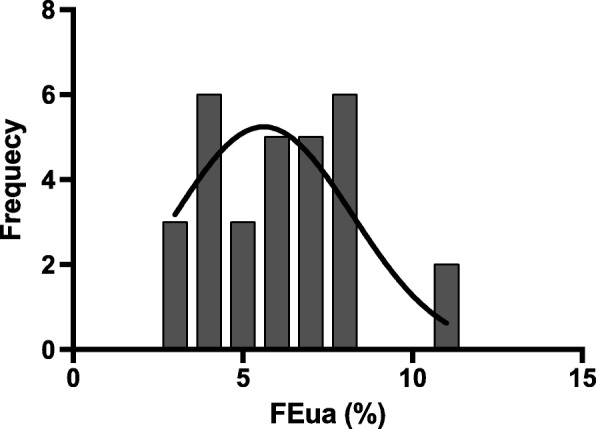
The distribution of FEua. The median value of FEua is approximately 6%

This study embraced 30 participants with the age of approximately 53.40 years and with 21 males (70.0%). Causes of renal injury in 30 CKD patients were showed (Fig. 2). Eight of them had no hypertension and history of antihypertensive medication. CKD patients with hypertension were treated with calcium-channel blockers, β blockers, angiotensin-converting enzyme inhibitors, angiotensin receptor blockers and selective α_1_ adrenergic receptor blockers, and there was no difference in the distribution of these drug between groups. The impression of data is shown in Table 1. Biochemical differences in two groups according to the median of FEua are also summarized in Table 1. Participants in the higher FEua group had higher Cur, EurGF, mMDP and nMDP than those in the lower FEua group (all *P* < 0.05, Table 1). The distribution pattern of FEua was presented (Fig. 3).

**Table 1:** Characteristics of the study population according to median of FEua

Variables	All (*n* = 30)	FEua1 (*n* = 15)	FEua2 (*n* = 15)	*P* value
		< 6.0%	≥6.0%	
Males gender (%)	70.00	66.7	73.30	0.500
Age(years)	53.50 (35.75, 58.25)	50.00 (31.00, 58.00)	55.00 (38.00, 59.00)	0.184
BMI (kg/m^2^)	24.33 (21.67, 26.33)	25.01 (21.30, 29.75)	24.16 (22.49, 25.44)	0.226
eGFR (ml/min/1.73 m^2^)	82.45 (69.08, 98.68)	81.60 (66.00, 99.80)	82.90 (71.50, 98.30)	0.298
SUA (umol/L)	366.30 ± 96.81	396.93 ± 94.99	335.67 ± 91.55	0.083
TC (mmol/L)	4.31 (3.95, 4.95)	4.50 (4.00, 5.05)	4.28 (3.71, 5.08)	0.215
TG (mmol/L)	2.10 (1.00, 2.80)	2.50 (1.33, 3.13)	1.80 (1.00. 2.25)	0.140
24-hUV(mL)	1631.78 ± 799.96	1610.03 ± 875.23	1653.52 ± 702.19	0.882
24-hUua(mmol)	3.18 ± 1.16	2.81 ± 1.10	3.56 ± 1.13	0.077
24-hUpro(mmol)	0.33 ± 0.66	0.47 ± 0.89	0.19 ± 0.28	0.255
Cur (ml/min/1.73 m^2^)	6.10 ± 2.04	5.09 ± 1.99	7.19 ± 1.49**	**0.003**
EurGF (mg/dL/1.73 m^2^)	21.21 ± 5.98	17.05 ± 3.45	25.37 ± 5.01**	**< 0.001**
Ccr (ml/min/1.73 m^2^)	103.62 ± 29.54	113.72 ± 32.37	92.79 ± 22.52	0.055
24 h MSP (mmHg)	124.00 (119.00, 132.50)	125.00 (119.00, 130.00)	124.00 (119.00, 140.00)	0.256
24 h MDP (mmHg)	77.07 ± 10.52	74.00 ± 9.64	80.13 ± 10.78	0.112
mMSP (mmHg)	127.82 ± 11.93	123.79 ± 11.11	131.86 ± 11.71	0.073
mMDP (mmHg)	79.86 ± 11.23	74.93 ± 8.78	84.79 ± 11.51*	**0.017**
dMSP (mmHg)	129.47 ± 13.71	127.33 ± 11.90	131.60 ± 15.43	0.195
dMDP (mmHg)	77.00 (72.50, 83.00)	77.00 (68.00, 82.00)	77.00 (74.00, 86.00)	0.173
nMSP (mmHg)	116.00 (110.00, 125.50)	115.00 (110.00, 123.00)	122.00 (110.00, 136.50)	0.101
nMDP (mmHg)	72.55 ± 10.99	68.00 ± 846	77.43 ± 11.55**	**0.0018**

*P < 0.05, **P < 0.01 vs. the FEua1 using the Independent-Sample Test (T-test) method if the variance is equal or the Tamhane’s T2 method when the variance is not equal, using Mann-Whitney U test in case of nonparametric data distribution. Numbers are mean ± SD or proportion. For age, BMI, eGFR, TC, TG, 24 h MSP, dMDP and nMDP, median and the 25th and 75th percentile are shown

*Abbreviations*: *BMI* Body mass index, *eGFR* Estimated glomerular filtration rate, *SUA* Serum uric acid, *TC* Total cholesterol, *TG* Triglycerides, *24-h UV* 24-h urinary volume, *24-h Uua* 24-h urinary uric acid, *24-h Upro* 24-h urinary protein, *Cur* Uric acid clearance rate, *EurGF* Excretion of uric acid per volume of glomerular filtration, *Ccr* Creatinine clearance, *24-h MSP* 24-h mean systolic pressure, *24-h MDP* 24-h mean diastolic pressure, *mMSP* Morning mean systolic pressure, *mMDP* Morning mean diastolic pressure, *dMSP* Day mean systolic pressure, *dMDP* Day mean diastolic pressure, *nMSP* Night mean systolic pressure, *nMDP* Night mean diastolic pressure

Boldface significant differences between FEua1 and FEua2

### Association of urinary uric acid excretion with blood pressure values

In order to study the association of the urinary uric acid excretion (FEua, Cur and EurGF) and 24-h blood pressure values (24-h MSP, 24-h MDP, mMSP, mMDP, dMSP, dMDP, nMSP, nMDP and CV of each period), all combinations are calculated after further permutations as shown below.

Pearson correlation analysis showed that FEua was positively associated with mMDP, nMDP and CV of dMSP (*r* = 0.408, *P* = 0.031; *r* = 0.415, *P* = 0.025; *r* = 0.413, *P* = 0.023, respectively), and not related to 24-h mean blood pressure values and other relevant time-period values, including 24-h MSP (*r* = 0.219, *P* = 0.244), 24-h MDP (*r* = 0.264, *P* = 0.159), CV of 24-h MSP and MDP (*r* = 0.253, *P* = 0.177; *r* = 0.200, *P* = 0.289), mMSP (*r* = 0.253, *P* = 0.194), CV of mMSP and mMDP (*r* = 0.007, *P* = 0.971; *r* = − 0.096, *P* = 0.628), dMSP (*r* = 0.132, *P* = 0.488), dMDP (*r* = 0.170, *P* = 0.370), CV of dMDP (*r* = 0.304, *P* = 0.102, nMSP (*r* = 0.328, *P* = 0.082), CV of nMSP and nMDP (*r* = 0.172, *P* = 0.373; *r* = 0.098, *P* = 0.613). There was a positive correlation between Cur and CV of dMSP (*r* = 0.408, *P* = 0.028).

Multiple linear regression analysis showed that FEua was still positively associated with the mMDP and nMDP after adjusting confounding factors [model 1 adjusted for age and BMI; model 2 plus gender and urine albumin to creatinine ratio (ACR); model 3 plus eGFR, TC and TG) (all *P* < 0.05, Table 2)]. However, the association of FEua and CV of dMSP disappeared (*P* > 0.05). Multiple linear regression analysis showed that Cur was still positively associated with CV of dMSP after adjusting confounding factors (model 1 adjusted for age and BMI; model 2 plus gender and ACR; model 3 plus eGFR, TC and TG) (all *P* < 0.05, Table 2).

**Table 2:** Multiple linear regression analysis for the association of mMDP and nMDP (independent variable) with FEua (dependent variable) and the association of CV of dMSP (independent variable) with FEua and Cur (dependent variable)

	Model 1			*AdjR*^*2*^	Model 2			*AdjR*^*2*^	Model 3			*AdjR*^*2*^
	*B*	*SE*	*P*		*B*	*SE*	*P*		*B*	*SE*	*P*	
FEua:												
mMDP	209.965	97.401	0.042	0.061	215.506	98.255	0.042	0.078	463.929	134.082	0.005	0.413
nMDP	214.828	93.775	0.031	0.128	273.428	99.687	0.013	0.229	416.231	153.804	0.019	0.313
CV of dMSP	0.762	0.338	0.033	0.074	0.914	0.334	0.013	0.113	1.154	0.550	0.056	0.125
Cur:												
CV of dMSP	0.08	0.004	0.037	0.067	0.009	0.004	0.033	0.032	0.012	0.004	0.011	0.297

Model 1 adjusted for age and BMI; model 2 plus gender and ACR; model 3 plus eGFR, TC and TG. Beta coefficients refer to how many deviations a dependent variable will change per deviation increase in the predictor variable. SE refers to standard error. The adjust *R*^2^ represents the goodness of fit, which is used to measure the degree of the estimated models fitting the observations

*Abbreviations*: *FEua* Fractional excretion of uric acid, *mMDP* Morning mean diastolic pressure, *nMDP* Night mean diastolic pressure, *CV of dMSP* Coefficient of variation of day mean systolic pressure, *Cur* Uric acid clearance rate, *BMI* Body mass index, *ACR* Albumin-to-creatinine ratio, *eGFR* Estimated glomerular filtration rate, *TC* Total cholesterol, *TG* Triglycerides

### Differences of mMDP, nMDP in FEua groups and CV of dMSP in Cur groups

**Fig. 4:**
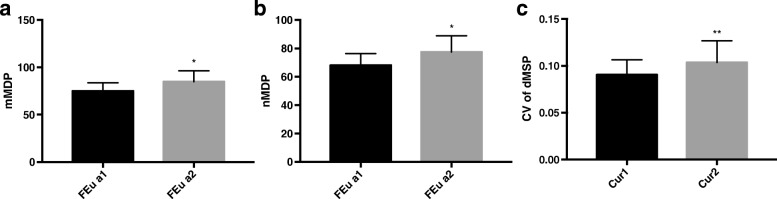
Differences of mMDP, nMDP in FEua groups and CV of dMSP in Cur groups. Levels of the values of mMDP, nMDP and CV of dMSP were compared according to the median of FEua by independent sample T test. With the higher FEua2 (FEua≥6.0%) group, the values of mMDP and nMDP went higher (both *P* < 0.05) than that of FEua1 (FEua<6.0%). Level of CV of dMSP was compared according to the median of Cur by independent sample T test. With the higher Cur2 (Cur ≥ 6.2 ml/min/1.73 m^2^) group, the values of CV of dMSP went higher (*P* < 0.01) than that of Cur1 (Cur < 6.2 ml/min/1.73 m^2^)

Compared with the higher FEua group, levels of mMDP and nMDP in the lower group (FEua< 6.0%) was lower than that in the higher FEua2 (FEua≥6.0%) group (both *P* < 0.05, Fig. 4a and b). Level of CV of dMSP in the higher Cur2 (Cur ≥ 6.2 ml/min/1.73 m^2^) group were higher than that in the lower Cur1 (Cur < 6.2 ml/min/1.73 m^2^) group (*P* < 0.01, Fig. 4c).

## Discussion

This study putted forward a comprehensive analysis of the association of urinary uric acid excretion with ambulatory BP values in CKD 1–2 patients. FEua and Cur, as markers for urinary uric acid excretion, were associated with the representative ambulatory blood pressure values. To the best of our knowledge, this study is the first to show a positive correlation of FEua with morning mean diastolic pressure and night mean diastolic pressure respectively.

Uric acid levels have been demonstrated to be correlated with blood pressure. According to our previous research [6], in the population with CKD and hypertension, fraction excretion of sodium (FEna) was negatively correlated with 24-h urinary uric acid and clearance of uric acid and positively correlated with FEua; urinary sodium/potassium ratio (Una/k) was negatively associated with 24-h Uua and Cur, and positively associated with FEua. To a large extent, these results suggest that the excretion of urinary sodium and potassium is closely related to the ability of the kidneys to process and excrete uric acid. So far what we know is that the excretion of urinary sodium and potassium is positively related to hypertension [12, 13]. In the previous basic study, we found that uric acid plays a down-regulated function in the NKA activity and its α1 subunit subcellular expression as well as the descending of the intracellular ATP, cell injury and the further activation of Src, NLRP3 and IL-1β [7]. Further, dysfunctional NKA caused by defected membrane of renal tubular cells result in diminished Na^+^ reabsorption and urinary concentration ability [14]. In other words, incremental urinary sodium excretion leads to hypertension [15]. All above is expected to prove that the extent of urinary uric acid excretion, whether it takes the form of FEua or Cur, may be related with the value of blood pressure.

Diastolic blood pressure is more closely related to uric acid levels. As we all know, systolic blood pressure is the pressure on the blood vessels when the heart contracts, while the diastolic blood pressure is generated by the elastic retraction of arteries. Therefore, diastolic blood pressure is more dependent on the elasticity and resistance of the blood vessel wall. In the Maastricht study [16], it is clear that serum uric acid and urinary uric acid excretion were associated with 24 h mean arterial pressure (MAP) but not with 24 h pulse pressure (PP). Similarly, diastolic blood pressure increases significantly as serum uric acid increases among children [17]. MAP is the steady component of BP reflecting vascular resistance, which can be increased by inhibiting the vasodilator nitric oxide [18, 19]. Reactive oxygen species (ROS) are produced during generation of uric acid. Xanthine oxidoreductase catalyzes the decomposition of xanthine into xanthine and xanthine into uric acid. When oxygen is an electron acceptor, superoxide radical anions (O^2−^) and hydrogen peroxide (H_2_ O_2_) are produced as by-products of the oxidation step [20]. These ROS directly decrease the bioavailability of the vasodilator nitric oxide and lead to the formation of peroxynitrite, which can increase the uncoupling of endothelial nitric oxide synthase, leading to more ROS. These changes are more intuitively reflected in the elasticity and resistance of blood vessels, which may explain why only diastolic blood pressure is elevated.

Some mechanisms may be responsible for the positive correlation of FEua with morning and night mean diastolic pressure, which had a special time range. Variations in blood pressure show a diurnal change, a surge in the morning and a decrease in sleeping time. The differences in blood pressure over the course of the day are due to circadian changes, and more specifically, behavioral changes in activity and rest (such as posture and meal times, psychological activity and sleeping), external environment (including ambient temperature and noise levels) and endogenous circadian rhythms in hemodynamic, endocrine, endothelial and neural variables, such as plasma noradrenaline and adrenaline levels (the autonomic nervous system), and renin, angiotensin and aldosterone levels (the renin–angiotensin–aldosterone system) [21–24]. RNA-seq analysis of mouse tissues revealed that the kidney is second only to the liver in the number of genes expressed in a circadian pattern [25]. Circulating levels of Ang II and aldosterone have a diurnal variation in humans, with higher plasma concentrations in the morning [26–28]. For the former, it binds to the correspond receptors to affect several systems and stimulates the blood pressure by constricting the blood vessels. Aldosterone, generated in the zona glomerulosa of adrenal cortex, plays a role in increasing the reabsorption of Na^+^ and secretion of K^+^. As we said before, the change of excretion of sodium and potassium is responsible for the increasing of blood pressure. Moreover, molecular clock in renal function plays a major role, which manifested in the renal excretion of water and major electrolytes [29]. What we already knew is that the excretion of uric acid is related to the major electrolytes in human body, including Na^+^ and K^+^. When dietary purine intake was kept constant, monophasic circadian fluctuations of fractional urate excretion (FEua) were observed with peak values in the afternoon, about 50% higher than during the night [30]. Due to the decreased excretion of uric acid in the nighttime, an upward trend may be shown in the serum uric acid. Thus, elevated serum UA levels can affect the development of rising blood pressure value [31]. Experimental studies have shown that raising serum UA can induce hypertension by stimulating oxidative stress, impairing endothelial function, and stimulating the renin angiotensin system [32, 33]. Furthermore, it also associated with morning blood pressure surge [34].

Cur, as a parameter to measure the uric acid removed from body, is positively related to CV of dMSP. In this research, the variability of blood pressure is not simply described as the standard deviation (SD) of the 24-h, morning, daytime and nighttime mean values obtained by using ambulatory BP monitoring, it was calculated as standard deviation (s.d.) divided by corresponding average BP called coefficient of variation of blood pressure (CV of BP). As proved in the prior study, SUA levels are independently associated with different BPV (the standard deviation of each time period) components in a population of newly diagnosed patients with essential hypertension [4]. With urinary uric acid excretion increased, SUA may fluctuate caused by dysfunction of renal handling of uric acid. As is discussed above, CV of dMSP is more sensitive to reflect the tiny fluctuations, especially during the active daytime.

This study had several potential limitations. First of all, the sample size is relatively small and there may be considerable deviations. Second, the values of blood pressure are generally not high, and some patients have accepted the antihypertensive treatment. Other limitations of this study include its cross-sectional design and one-time ABPM. Since the study explored the correlation between urinary uric acid excretion with blood pressure, antihypertensive treatment will affect the results of the observation and detection.

## Conclusion

In summary, we demonstrated that there is a positive correlation of FEua with morning and night mean diastolic pressure separately and Cur is positively related to CV of dMSP in CKD population. Monitoring the trend of urinary uric acid, may have a role in the early detection for hypertension or relative risks in the population of CKD. More researches are needed to elucidate mechanism underlying higher urinary uric acid excretion leading to hypertension.

## Data Availability

The datasets generated and analyzed during the current study are not publicly available due patient privacy but are available from the corresponding author on reasonable request.

## Abbreviations

ABPM: Ambulatory BP monitoring
ACR: Urine albumin to creatinine ratio
BMI: Body mass index
BPV: Blood pressure variability
BUN: Urinary nitrogen
CKD: Chronic kidney disease
Cur: Uric acid clearance rate
CV of dMSP: Coefficient of variation of day mean systolic pressure
eGFR: Estimated glomerular filtration rate
EurGF: Excretion of uric acid per volume of glomerular filtration
FBG: Fasting blood glucose
FEna: Fraction excretion of sodium
FEua: Fractional excretion of uric acid
HDL: High-density lipoprotein
HTN: Hypertension
IQR: Medians with inter quartile range
LDL: Low-density lipoprotein
LSD: Least significance difference
MAP: Mean arterial pressure
MBPS: Morning blood pressure surge
mMDP: Morning mean systolic pressure
NKA: Na^+^-K^+^-ATPase
nMDP: Night mean diastolic pressure
PP: Pulse pressure
ROS: Reactive oxygen species
Scr: Creatinine
SD: Standard deviation
SUA: Uric acid
TC: Total cholesterol
TG: Triglycerides
Una/k: Urinary sodium/potassium ratio
